# Supplementary material for: Pax-5 Protein Expression Is Regulated by Transcriptional 3′UTR Editing
Source: Cells. 2021 Dec 28;11(1):76. doi: 10.3390/cells11010076 (PMC8750734; doi:10.3390/cells11010076)
Supplement: Supplementary file 1 [file cells-11-00076-s001.zip › cells-1490372-supplementary.pdf]

Supplementary Table S1: Oligonucleotide primer sequences

Primers	**Forward primer** (5'-3')	**Reverse primer** (5'-3')
Pax5 spliced region	TACAACGACCCCTGGAGGTT	TTCCACTCCAAGGGAAATGGG
Pax5 nested spliced region	GGGAAATGCGCCGTTTGTAA	AGCCCTCCTGAAGATAGCCA
Pax-5 Gene specific 5'	AGTGAGTTTTCCGGGAGTCCCTA	
Pax-5 Nested Gene specific 5'	TCGTACAACGACTCCTGGAGGTT	
MCF10A DNA walk 2.5kb	CAAACACTGATGGCACCTATTG	GATGCCAGGTAACATACCTTCT
MCF10A DNA walk 3kb	CAAACACTGATGGCACCTATTG	CCTTTGGAGCCATCCTCAC
MCF10A DNA walk 3.5kb	CAAACACTGATGGCACCTATTG	CCTGTTCTTTCTTGCCTGATTG
MCF10A DNA walk 4kb	TGGGTCTCTGCAAACCAATAG	GGACAGGAGGTGGATGAGAAG
Puromycin	GGCCTTCCATCTGTTGCT	TGCAAGAACTCTTCCTCACG

Supplementary Table S2: *In-silico* analysis of polyadenylation signals within the *Pax-5* 3'UTR

**Polyadenylation prediction site software**	**Predicted polyA signals** (motif position in nucleotides following stop codon)
**Softberry POLYAH***<br>http://www.softberry.com/berry.phtml?topic=polyah&group=programs&subgroup=promoter	4912 (AATAAA)<br>6015 (AATAAA)<br>6019 (AATAAA)
**DNA polyA signal miner****<br>http://dnafsminer.bic.nus.edu.sg	4912 (AATAAA)<br>6015 (AATAAA)<br>6019 (AATAAA)<br>7262 (AATAAA)<br>4059 (AAGAAA)<br>3500 (AAGAAA)<br>766 (AAGAAA)
**PolyA pred****<br>http://crdd.osdd.net/raghava/polyapred/index.html	4912 (AATAAA)<br>6019 (AATAAA)<br>766 (AAGAAA)<br>4059 (AAGAAA<br>3902 (AATGAA)<br>255 (AATAGA)<br>1706 (AGACAA)<br>2838 (AGACAA)<br>3230 (ACCTTA)

* Softberry prediction software enables searches of canonical polyA consensus motifs (Salamov et al., 1997)
**DNA polyA miner (H. Liu et al., 2005) and PolyA pred (Ahmed et al., 2009) enable searches of canonical and non-canonical polyA motifs.

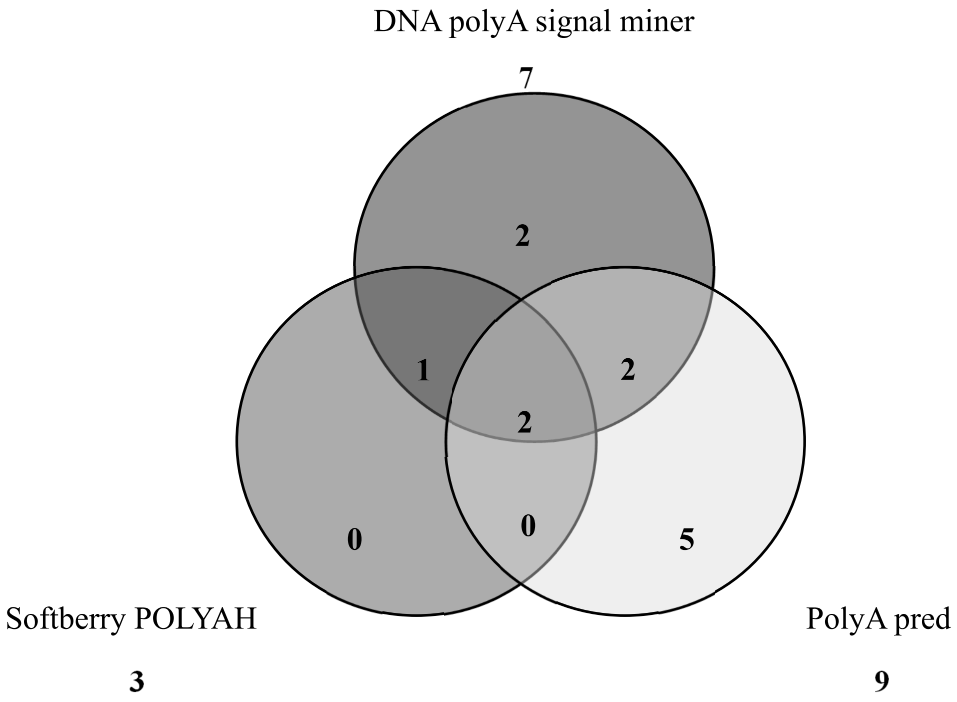

<u>Supplementary Figure S1</u>: Prediction of putative polyA signals in the *Pax-5* 3'UTR. Putative poly-A signals encoded within the *Pax-5* 3'UTR were identified using computational polyA prediction tools (http:// dnafsminer.bic.nus.edu.sg; http://crdd.osdd.net/raghava/polyapred/index.html; and, http://www.softberry.com/berry.phtml?topic=polyah&group=programs&subgroup=promoter). The results were then compared using a Venn diagram to decipher common sites from different software tools. Two canonical polyA sites (located at 6019 nt and 4912 nt) were detected by all three programs whereas, the two non-canonical polyA sites (located at 766 nt and 4059 nt) were identified by "*DNA polyA miner*" and "*PolyApred*". The others remain potential non-canonical polyA sites.

Supplementary Table S3: Predicted miRNA targeting of the *Pax-5* 3'UTR.

***In silico* Prediction tool**	**miRNAs**	**miRNA target positions after stop codon** (nt)	**Score**	**Reported miRNA functions** (ref.)
**microRNA.org***	hsa-miR-133a, b	1881	-0.4207	Tumor suppressor (Patron et al., 2012; Y. Zhao et al., 2014)
	hsa-miR-181a, b, c, d	830	-0.2561	Tumor suppressor (de Yebenes et al., 2008; Lwin et al., 2010).
	hsa-miR-376b	200	-0.2371	Involved in neoplastic transformation (Korkmaz et al., 2012)
	hsa-miR-875-5p	806	-0.2182	Uncharacterized
	hsa-miR-217	712	-0.1493	Acts as tumour suppressor in many cancers including leukemia (Nishioka et al., 2014)
	hsa-miR-328	1748	-0.1202	Down regulated in chronic myelogenous leukemia (Eiring et al., 2010).
	hsa-miR-33a, b	1205	-0.1154	Expressed in many tissues including hematopoietic system (Landgraf et al., 2007).
	hsa-miR-23a, b	836	-0.1159	Implicated in hypoxia in breast cancer (Camps et al., 2014)
	has-mir-433	199	-0.2521	Drug resistance and down regulated in solid tumors (Luo et al., 2009; Weiner-Gorzel et al., 2015)
	hsa-miR372	776	-0.2219	Roles differ depending on cancer tissues.
**miRDB****	hsa-miR-4447	1046, 2038, 2336, 3424, 3732, 5677	97	Uncharacterized
	hsa-miR-1275	1045, 1552, 2037, 3423, 3447, 3731	93	Breast cancer progression (Pena-Chilet et al., 2014)
	hsa-miR-1227-5p	542, 2231, 3362, 4724, 6272, 6330	90	Uncharacterized
	hsa-miR-4516	191, 5758	85	Targets oncogenic pathways (Chowdhari et al., 2014)
	hsa-miR-1285-5p	7183	79	Tumor suppressor (Hidaka et al., 2012; J. Liu et al., 2015)
	hsa-miR-4472	77, 1046, 2038, 2336, 3424, 3732, 5677	69	Uncharacterized
	hsa-miR-548e-5p	328, 368, 6527, 6849	68	Uncharacterized
	hsa-miR-6867-5p	2875, 4797, 6077, 6085, 6093, 6101	68	Uncharacterized
	hsa-miR-6865-3p	4630, 6295	66	Uncharacterized
	hsa-miR-221-5p	1406, 2830, 6505	64	Up regulated in clinical DLBCL. Also implicated drug resistance in breast cancer (Lawrie et al., 2007; Wei et al., 2014)
	hsa-miR-4531	3052, 5759	64	Uncharacterized
	hsa-miR-8073	1406, 2830, 6505	64	Uncharacterized
	hsa-miR-185-3p	248, 892, 3832, 4953, 4962, 6342	63	Overexpressed in subtypes of lymphoma (Jima et al., 2010)

	hsa-miR-574-5p	6107	62	Overexpressed and oncogenic (Yang et al., 2013)
TargetScan***	hsa-miR-137	7127	0,88	Involved in neoplastic transformation (Mahmoudi et al., 2016)
	hsa-miR-206	5966	0,86	Tumor suppressor in cancers (Nohata et al., 2012)
	hsa-miR-1-3p	5966	0,86	Tumor suppressor in cancers (Weiss et al., 2016)
	hsa-miR-613	5966	0,86	Tumor suppressor in cancers (Wu et al., 2016)
	hsa-miR-1271-5p	6919, 6928	0,84	Tumor suppressor in cancers (H. Liu et al., 2016)
	hsa-miR-9-5p	6930	0,83	Involved in neoplastic transformation (X. Liu et al., 2016)
	hsa-miR-218-5p	2871	0,8	Involved in neoplastic transformation (Lu et al., 2015)
	hsa-miR-92b-3p	7126	0,79	Tumor suppressor in cancers (G. Ma et al., 2016)
	hsa-miR-182-5p	6927	0,79	Role in neoplastic transformation (Y. Ma et al., 2016; F. Wang et al., 2015)
	hsa-miR-181-5p	6902	0,75	Tumor suppressor in cancers (de Yebenes et al., 2008; Lwin et al., 2010).
	hsa-miR-96-5p	6928	0,74	Role in neoplastic transformation (Y. Ma et al., 2016; Wang et al., 2016)
	hsa-miR-194-5p	7176	0,67	Involved in neoplastic transformation (Gu et al., 2013)
	hsa-miR-138-5p	3052, 4096, 5218	0,63	Tumor suppressor in cancers (Jin et al., 2013)
	hsa-miR-183-5p.1	7238	0,53	Tumor suppressor in cancers (Y. Ma et al., 2016)
	hsa-miR-140-5p	3287, 2842	0,49	Involved in neoplastic transformation (Green et al., 2015)
	hsa-miR-223-3p	6836, 3256	0,49	Involved in neoplastic transformation (Y. Pan et al., 2014)
	hsa-miR-455-3p.2	7088, 6595	0,38	Tumor suppressor in cancers (J. Liu et al., 2016)
	hsa-miR-148b-3p	6188, 4432	0,25	Involved in neoplastic transformation (Y. Chen et al., 2013)
	hsa-miR-4770	5351, 1449	0,24	Uncharacterized
	hsa-miR-6088	1449, 5351	0,24	Uncharacterized
	hsa-miR-143-3p	1449, 5351	0,24	Involved in neoplastic transformation (Y. Pan et al., 2014)

*This database (Betel et al., 2008) uses an old *Pax-5* reference sequence version number (NM_016734.1) in NCBI July 2013 which is characterized by a much shorter 3'UTR (2026 nt). The score cut off was set at -0.1 or lower for adequate prediction. (http://www.microrna.org/)

**This database (Y. Chen et al., 2020) uses new reference gene versions in NCBI March 2015 (NCBI/NM_016734.3) (W. Zhao et al., 2019). The length equals 7285 nt. A score greater than 60 is an adequate prediction. (http://mirdb.org/)

***This database (Agarwal et al., 2015) uses a new version 7.2 released in March 2018 for miRNA prediction sites on a 7285 nt *Pax-5* 3'UTR. http://www.targetscan.org/vert_72/

Supplementary Table S4: Clinical data relating to patient samples

**Patient ID** (code)	**DOB** (dd/mm/yy)	**Age** (yrs)	**Gender**	**Clinical diagnosis**	**Grading**	**Staging***
041	3/06/52	61	M	CLL		Rai 0-1
020	9/04/35	77	M	CLL		Rai-1
026	5/12/48	63	F	CLL		Rai-1
038	8/06/51	61	F	CLL		Rai-1
032	8/03/36	76	M	DLBCL		IA
007	1/03/63	49	M	FL	2	IIA
016	31/12/45	66	M	DLBCL		IIA
019	21/09/44	67	M	FL	2	IIAE
021	8/12/49	62	F	DLBCL		IIB
011	2/04/37	74	F	FL	1/2	IIIA
028	14/01/48	64	M	B55		IIIA
029	17/11/36	76	F	FL	3a/3	IIIA
033	20/05/71	41	M	HL (NS)		IIIB
014	3/11/53	58	M	SLL		IVA
025	31/05/56	56	F	MZL		IVA
027	5/02/49	63	F	FL		IVA
002	22/08/64	47	M	DLBCL		IVB

"

* Cancer staging for disease progression is based on the TNM (Tumor/Node/Metastasis) and Rai (for CLL) staging systems. Hematopoietic cancer diagnoses are: Chronic lymphocytic leukemia (CLL); Diffused large B-cell lymphoma (DLBCL); Follicular lymphoma (FL); Small lymphocytic lymphoma (SLL); Hodgkin lymphoma (HL) with nodular sclerosis (NS); and Marginal zone lymphoma (MZL).
